# Development and Validation of a Novel Prognostic Model for Overall Survival in Newly Diagnosed Multiple Myeloma Integrating Tumor Burden and Comorbidities

**DOI:** 10.3389/fonc.2022.805702

**Published:** 2022-03-17

**Authors:** Shuangshuang Jia, Lei Bi, Yuping Chu, Xiao Liu, Juan Feng, Li Xu, Tao Zhang, Hongtao Gu, Lan Yang, Qingxian Bai, Rong Liang, Biao Tian, Yaya Gao, Hailong Tang, Guangxun Gao

**Affiliations:** Department of Hematology, Xijing Hospital, Air Force Medical University, Xi’an, China

**Keywords:** prognostic model, risk stratification, HCT-CI, comorbidity, multiple myeloma

## Abstract

**Background:**

Multiple myeloma (MM) is a highly heterogeneous disease with enormously variable outcomes. It remains to be a major challenge to conduct a more precise estimation of the survival of MM patients. The existing stratifications attached less importance to the prognostic significance of comorbidities. In the present study, we aimed to develop and validate a novel and simple prognostic stratification integrating tumor burden and comorbidities measured by HCT-CI.

**Method:**

We retrospectively enrolled 385 consecutive newly diagnosed multiple myeloma (NDMM) patients in Xijing Hospital from January 2013 to December 2020. The cohort between January 2016 and December 2020 was selected as development cohort (*N* = 233), and the cohort between January 2013 and December 2015 was determined as validation cohort (*N* = 152). By using LASSO analysis and univariate and multivariable Cox regression analyses, we developed the MM-BHAP model in the way of nomogram composed of β2-MG, HCT-CI, ALB, and PBPC. We internally and externally validated the MM-BHAP model and compared it with ISS stage and R-ISS stage.

**Results:**

The MM-BHAP model was superior to the ISS stage and partially better than the R-ISS stage according to time-dependent AUC, time-dependent C-index, DCA, IDI, and continuous NRI analyses. In predicting OS, only the MM-BHAP stratification clearly divided patients into three groups while both the ISS stage and R-ISS stage had poor classifications in patients with stage I and stage II. Moreover, the MM-BHAP stratification and the R-ISS stage performed well in predicting PFS, but not for the ISS stage. Besides, the MM-BHAP model was also applied to the patients with age ≤65 or age >65 and with or without HRCA and could enhance R-ISS or ISS classifications.

**Conclusions:**

Our study offered a novel simple MM-BHAP stratification containing tumor burden and comorbidities to predict outcomes in the real-world unselected NDMM population.

## Introduction

Multiple myeloma (MM) is the second most common hematologic malignancy characterized by hyperproliferation of clonal plasma cells within the bone marrow (1). Despite its incurability, the median overall survival (OS) in MM patients has obtained a significant improvement over the years due to the introduction of novel therapies, such as proteasome inhibitors (PIs), immunomodulatory drugs (IMIDs), monoclonal antibodies, and immunotherapy. However, even among the patients with the same genetic background currently known, the high heterogeneity of the outcome still exists, with the survival varying from a few months to more than 10 years (2–4). Therefore, it remains to be a major challenge to conduct precise estimation of the survival in MM patients.

The current International Staging System (ISS) (5) and Revised International Staging System (R-ISS) (6) were widely used in clinical practices. The R-ISS stage combined the ISS stage, lactate dehydrogenase (LDH), and high-risk chromosomal abnormalities (HRCA), which achieved an improvement in estimating prognosis compared to the ISS stage. However, the R-ISS was composed of disease-related factors, which mainly reflected the inherent biological characteristics of myeloma but reckoned without the patient-related factors like comorbidities and performance status. Moreover, there was a large portion of patients distributed into R-ISS stage II (6), indicating that it was necessary to conduct a more precise estimation of prognosis for better differentiation in these patients. Besides, the R-ISS stage was based on the data derived from clinical trials and might result in some limitations for the application in the real-world unselected patients.

Based on the abovementioned reasons, efforts were always underway to explore new risk stratifications for the prediction of survival in the patients with newly diagnosed multiple myeloma (NDMM). Some studies established prognostic tools with biological parameters, such as gene expression (7–14) and lncRNA (15), followed by the limitations of the complexity and non-standardization. A few of new prognostic models predicting survival were based on new integration of chromosomal abnormalities and clinical indicators, whereas these only focused on disease-related factors (2, 16–18). Therefore, a more comprehensive assessment, orchestrating the genetic landscape of myeloma and host characteristics, may be more appropriate to distinguish the benefits from existing treatments in an unselected community setting. For example, the UK Myeloma Research Alliance Risk Profile (MRP) taking account of WHO performance status and age, together with ISS stage and C-reactive protein, could help to predict the prognosis and therapy delivery in patients who are not candidates for transplantation (19). Furthermore, a survival matrix was created using the factors of age, del (17p), triplet therapy use, EQ-5D mobility, ISS stage, solitary plasmacytoma, history of diabetes, platelet count, Eastern Cooperative Oncology Group performance status, and serum creatinine for predicting the outcomes of NDMM patients (20). Moreover, a pleural effusion-based nomogram including factors of pleural effusion, plasma cell proportion in the bone marrow, ISS stage, Charlson Comorbidity Index (CCI), 1q21 gain, and autologous hematopoietic stem cell transplantation (HSCT) (21) was also performed to evaluate the prognosis in unselected MM population.

It is obvious that there is still a lack of comprehensive studies for the prognostic significance of comorbidities which are usually excluded from the clinical trials but maybe partially determine the treatment options, treatment intensity, and time to next treatment (3, 4). Both the CCI and hematopoietic cell transplantation-comorbidity index (HCT-CI) were ways of evaluating the impact of comorbidities. However, the HCT-CI could have clearer definitions of comorbidities than the CCI (22) and also considered recent infections which usually led to less intensive therapies and associated with worse OS (23). Moreover, HCT-CI was widely used to predict the survival probabilities of patients after HSCT (24–27), while little attention was paid to its prognostic value in the outcome of NDMM patients.

Hence, we explored the possibility of predicting the survival of MM patients with the HCT-CI evaluated at the time of diagnosis. Using routinely available clinical factors that integrated comorbidities and tumor burden, we developed and validated a new prognostic model and risk stratification for predicting the probability of 6-month, 1-year, 2-year, and 4-year OS of patients with NDMM. Our study offered a novel simple tool to predict outcomes in NDMM patients, as a supplement for a better stratification on the basis of current risk classifications.

## Methods

### Cohort Selection

We retrospectively enrolled 385 consecutive newly diagnosed MM patients in our institution from January 2013 to December 2020. All patients were aged at least 18 years, diagnosed according to International Myeloma Working Group (IMWG) criteria (28) and followed up for the available treatment and survival information until June 1, 2021. The cohort between January 2016 and December 2020 was selected as development cohort (*N* = 233), and the cohort between January 2013 and December 2015 was determined as validation cohort (*N* = 152).

### Data Collection

The baseline characteristics, such as patient/disease-specific data at diagnosis and treatment information, were collected. The patient-specific data included age, sex, body mass index (BMI, kg/m^2^), history of hypertension, history of thrombosis, and HCT-CI. The disease-specific data contained white blood cell (WBC, ×10^9^/L), neutrophil granulocyte (NEU, ×10^9^/L), lymphocyte (LYM, ×10^9^/L), monocyte (MONO, ×10^9^/L), hemoglobin (HGB, g/L), platelet (PLT, ×10^9^/L), β2-microglobulin (β2-MG, mg/L), albumin (ALB, g/L), serum calcium (Ca^2+^, mmol/L), lactate dehydrogenase (LDH, IU/L), high-risk chromosomal abnormalities (HRCA), bone marrow plasma cells (BMPC), peripheral blood plasma cells (PBPC), DS stage, ISS stage, and R-ISS stage. The treatment information involved novel therapies and autologous stem-cell transplantation (ASCT).

### Definition of Some Variables and Survival Outcomes

The condition of infection in HCT-CI was assessed at the time before induction therapy and other comorbidities that were measured at diagnosis in our study. The HRCA was defined as the presence of t (4;14) and/or t (14;16) and/or del(17p), detected by fluorescence *in situ* hybridization (FISH) (6). The thresholds were set at 10% for t (4;14) and t (14;16) and at 20% for del(17p), recommended by the European Myeloma Network (29). The BMPC was referred to as the proportion of clonal plasma cells on a bone marrow smear. The PBPC meant the proportion of clonal plasma cells on a peripheral blood smear.

The time of last follow-up was on June 1, 2021. The primary end point was OS defined as the time from the start of diagnosis until all-cause death or until the last follow-up time the patient was known to be alive. The secondary end point was progression-free survival (PFS) defined as the time from the start of diagnosis until progression or all-cause death or until the last follow-up time the patient was known to be progression-free.

### Development of the New Prognostic Model

Before variable selection, we examined the non-linear association between continuous variables with OS *via* restricted cubic splines based on Cox regression (**Supplemental Figure S1**), then transformed the variable into categorical variable according to the cutoff points when the *p* value for non-linearity <0.05. We handled missing data on candidate prognostic variables using multivariate imputation by chained equation (MICE) and created five imputed datasets. Then, we evaluated the potential prognostic value of candidate variables by using univariate Cox regression analysis in all the five imputed datasets and the coefficients were combined with R package “mice.” The variables with *p* value < 0.10 were subjected to the least absolute shrinkage and selection operator (LASSO) analysis to prevent overfitting (30), and we finally picked up four predictors of them taking account of clinical importance and prediction of the model (**Supplemental Methods**).

### Validation of the New Prognostic Model

We internally and externally validated the predictive power of the model respectively in the development cohort and validation cohort *via* the following analyses: (1) discrimination: assessed by time-dependent area under the curve (AUC) of the receiver operator characteristic (ROC) and time-dependent Harrell’s concordance index (C-index) analyses; (2) calibration: examined by calibration curve with 1,000 bootstrap resamples, which indicated a good fit of the predicted probabilities with actual outcome frequencies when the curve had a good agreement with the 45° diagonal line; (3) clinical usefulness: estimated by decision curve analysis (DCA), which showed that the model was the best choice for all patients that had the highest net benefits among all the range of risk thresholds (31); and (4) improvement in prediction: tested by integrated discrimination improvement (IDI) and continuous net reclassification index (NRI), which suggested that the new model had an improvement in predictive capacity compared with the old model when they were greater than zero. In particular, we would like to compare the performance of the new prognostic model with that of the R-ISS stage which showed missing data in the development cohort. To reduce the influence of data deficiency, we carried out the abovementioned analyses in each imputed dataset.

### Statistical Methods

All the statistical analyses were carried out using R version 4.1.0 and SPSS version 26.0, and a two-sided *p* < 0.05 suggested a statistical significance. The chi-square test or Fisher’s exact test was used to analyze qualitative variables, and the Mann–Whitney U test was used to analyze quantitative variables. Survival was analyzed by Kaplan–Meier curves and log-rank tests. Uni- and multivariable Cox proportional hazard models were used to assess the prognostic factors and calculated hazard ratios (HR) with 95% confidence intervals (CI). The R packages used in the abovementioned analyses were shown in **Supplemental Methods**.

## Results

### Patients’ Characteristics

We presented the baseline characteristics of the development cohort (*N* = 233) and validation cohort (*N* = 152) in the **Supplemental Table S1**. Moreover, the median age was respectively 59 (35–88) and 58.5 (18–89) years. In the development cohort, the vast majority (99.6%) of the patients received anti-myeloma treatment and most (98.3%) patients underwent novel therapy in their induction treatment. Similarly, 98.7% of the patients were treated with anti-myeloma therapy and 98.0% of the patients received novel therapy in the validation cohort. As of the end of June 1, 2021, the median time of follow-up was 24.0 (range from 0.1 to 61.8) and 28.8 (range from 0.4 to 101.9) months in the development cohort and validation cohort, respectively.

### Development and Evaluation of the MM-BHAP Model

**Figure 1:**
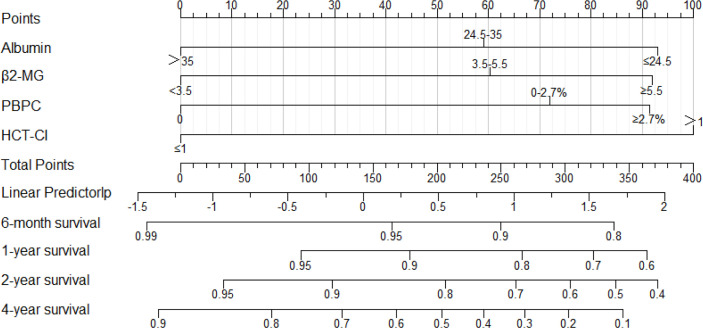
The nomogram derived from the development cohort to predict OS of NDMM patients. The precise values of each variable are showed in **Supplemental Table S2**. Also, the likelihood of 6-month, 1-year, 2-year, or 4-year survival was predicted according to the total points which are located on the corresponding axes.

In the development cohort, we evaluated the correlation of candidate variables with OS using univariate Cox regression analysis (**Table 1**), and the variables with *p* value < 0.10 were subjected to the LASSO analysis. Finally, we picked up four variables to construct a new prognostic model in the way of nomogram (**Figure 1** and **Supplemental Table S2**) by using the multivariate Cox proportional hazard model based on the β coefficients of predictive factors (**Table 2**). The nomogram consisted of β2-MG, HCT-CI, ALB, and PBPC, which comprised a new prognostic model called MM-BHAP model for predicting the probability of 6-month, 1-year, 2-year, and 4-year OS in NDMM patients. Also, we constructed two additional models individually including only ISS stage or R-ISS stage, comparing the predictivity of the MM-BHAP model with them in each imputed dataset (we just showed the results in one of the five imputed datasets in the text due to their similarity).

**Table 1:** Univariate Cox regression analyses in the development cohort.

Characteristics	HR	95% CI	*p*
**Age >65 vs. ≤65 years**	1.64	0.91–2.95	0.098
**Male (vs. female)**	1.37	0.74–2.56	0.32
**BMI (kg/m^2^)**			
** >22.5 and ≤25.5 vs. ≤22.5**	0.92	0.47–1.79	0.803
** >25.5 vs. ≤22.5**	0.69	0.28–1.71	0.410
**History of hypertension**			
** Yes vs. no**	0.91	0.45–1.83	0.788
**History of thrombosis**			
** Yes vs. no**	0.9	0.36–2.28	0.827
**HCT-CI >1 vs. ≤1**	2.46	1.39–4.33	0.002
**WBC >8.85 vs. ≤8.85 ×10^9^/l**	2.28	1.07–4.88	0.034
**Neu (×10^9^/L)**	1.18	1.05–1.34	0.008
**LYM (×10^9^/L)**	1.35	1.07–1.72	0.013
**MONO >0.9 vs. ≤0.9 ×10^9^/L**	4.25	1.68–10.75	0.002
**HGB (g/L)**			
**>70 and ≤120 vs. ≤70**	0.68	0.33–1.39	0.29
**>120 vs. ≤70**	0.41	0.16–1.09	0.074
**PLT >228 vs. ≤228 ×10^9^/L**	0.69	0.31–1.54	0.369
**β2-MG (mg/L)**			
**≥3.5 and <5.5 vs. <3.5**	1.48	0.51–4.26	0.471
**≥5.5 vs. <3.5**	3.78	1.58–9.06	0.003
**ALB (g/L)**			
**>24.5 and ≤35 vs. ≤24.5**	0.60	0.24–1.47	0.263
**>35 vs. ≤24.5**	0.30	0.12–0.77	0.012
**Serum calcium (mmol/L)**	2.36	0.90–6.17	0.079
**LDH >300 vs. ≤300 IU/L**	1.57	0.52–4.67	0.391
**HRCA: yes vs. no**	1.98	0.89–4.43	0.089
**BMPC >61.7% vs. ≤61.7%**	1.54	0.78–3.04	0.211
**PBPC**			
**>0 and <2.7% vs. 0**	2.07	0.91–4.70	0.082
**≥2.7% vs. 0**	3.20	1.51–6.78	0.002
**DS stage**			
** II vs. I**	0.64	0.14–2.9	0.64
** III vs. I**	1.83	0.57–5.92	0.314
**ISS stage**			
** II vs. I**	0.73	0.24–2.27	0.589
** III vs. I**	3.33	1.40–7.93	00.007
**R-ISS stage**			
** II vs. I**	1.23	0.40–3.77	0.707
** III vs. I**	5.14	1.75–15.09	0.004
**ASCT: yes vs. no**	0.29	0.07–1.21	0.09

**Table 2:** Multivariate Cox regression analysis of variables included in the MM-BHAP model in the development cohort.

Characteristics	Coefficient	HR		95% CI	*p*
**ALB (g/L)**					
**>24.5 and ≤35 vs. ≤24.5**	-0.29		0.75	0.29–1.91	0.546
**>35 vs. ≤24.5**	-0.79		0.45	0.17–1.23	0.121
**β2-MG (mg/L)**					
**≥3.5 and <5.5 vs. <3.5**	0.51		1.67	0.57–4.86	0.346
**≥5.5 vs. <3.5**	0.78		2.18	0.85–5.58	0.100
**PBPC (%)**					
**>0 and <2.7 vs. 0**	0.61		1.84	0.78–4.38	0.165
**≥2.7 vs. 0**	0.78		2.18	0.96–4.93	0.062
**HCT-CI (points)**					
**>1 vs. ≤1**	0.85		2.34	1.22–4.49	0.010
**Statistical analysis of the prognostic model**					
**Likelihood ratio test**					<0.001
**Wald test**					<0.001
**Score (log-rank) test**					<0.001

**Figure 2:**
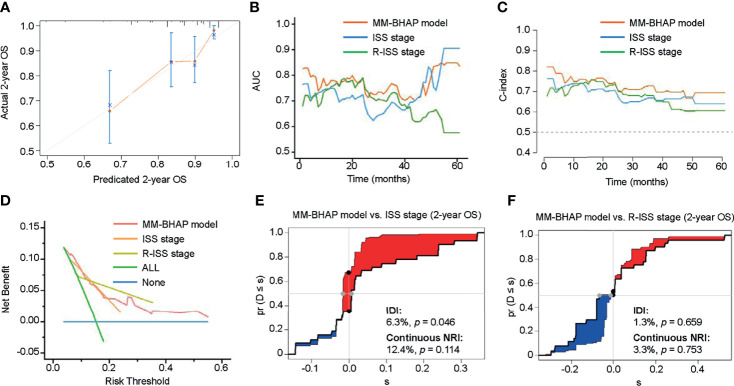
The performance of the MM-BHAP model, ISS stage, and R-ISS stage for predicting OS in the development cohort. **(A)** The calibration curve of the MM-BHAP model for predicting 2-year OS. **(B)** The time-dependent AUC of the ROC in the three models. **(C)** The time-dependent Harrell’s C-index in the three models. **(D)** The DCA was used to estimate clinical usefulness of the three models for predicting 2-year OS. The improvement in prediction of the MM-BHAP model was compared to the ISS stage **(E)** or R-ISS stage **(F)**. The IDI was the value of the difference in area between red and blue zones **(E, F)**. The continuous NRI was the value of the distance between two black dots **(E, F)**.

In the development cohort, the 50-sample bootstrapped calibration curve, with 1,000 bootstrap resamples, was used to examine the calibration of the MM-BHAP model. **Table 3** summarizes other evaluations of models. The predictive OS probabilities were basically in accordance with those observed in 2-year OS (**Figure 2A**). As for discrimination, both time-dependent AUC and C-index of MM-BHAP model were globally higher than that of ISS stage and R-ISS stage (**Figures 2B, C** and **Table 3**). We also assessed clinical effect by DCA for 2-year OS (**Figure 2D**). The MM-BHAP model could achieve positive net benefit over a wider range of risk threshold, with higher area under the decision curve analysis (AUDC) than ISS stage and R-ISS stage in most time-points (**Table 3**). Moreover, the results of calibration curve and DCA for 6-month, 1-year, and 4-year OS are shown in **Supplemental Figure S3**.

**Table 3:** Comprehensive evaluations of the different models in the development cohort.

OS	6 months	12 months	24 months	48 months
**AUC, n (95% CI)**				
**MM-BHAP**	0.793 (0.691–0.895)	0.781 (0.684–0.878)	0.789 (0.702–0.875)	0.721 (0.566–0.875)
**ISS stage**	0.720 (0.626–0.815)	0.749 (0.686–0.813)	0.724 (0.644–0.803)	0.771 (0.647–0.896)
**R-ISS stage**	0.730 (0.590–0.871)	0.755 (0.655–0.856)	0.791 (0.696–0.886)	0.636 (0.527–0.746)
**C-index, n**				
**MM-BHAP**	0.788	0.760	0.724	0.667
**ISS stage**	0.720	0.732	0.680	0.670
**R-ISS stage**	0.714	0.736	0.722	0.600
**Range*^a^*, n (%)**				
**MM-BHAP**	1.25%–26.64%	2.28%–38.16%	3.78%–61.25%	11.51%–95.07%
**ISS stage**	2.05%–8.99%	3.69%–15.74%	5.88%–24.12%	16.42%–55.84%
**R-ISS stage**	2.68%–13.29%	4.84%–22.97%	8.01%–35.53%	25.23%–78.31%
**AUDC, n**				
**MM-BHAP**	0.0027	0.0094	0.0195	0.0757
**ISS stage**	0.0015	0.0052	0.0106	0.0472
**R-ISS stage**	0.0018	0.0058	0.0144	0.0922
**IDI, n (95% CI), *p* value**				
**Vs. ISS stage**	2.4% (-0.3%–14.8%) *p* = 0.088	3.9% (-0.4%–19.5%) *p* = 0.080	6.3% (1.0%–22.1%) *p* = 0.046	-1.8% (-23.1%–24.7%) *p* = 0.815
**Vs. R-ISS stage**	0.6% (-3.7%–11.3%) *p* = 0.547	1.1% (-5.4%–16.4%) *p* = 0.500	1.3% (-13.1%–15.3%) *p* = 0.659	0% (-26.9%–34.5%) *p* = 0.685
**Continuous NRI, n (95% CI), *p* value**				
**Vs. ISS stage**	1.2% (-14.9%–48.6%) *p* = 0.314	-2.1% (-16.1%–41.9%) *p* = 0.596	12.4% (-4.4%–50.2%) *p* = 0.114	-21.1% (-82.1%–70.9%) *p* = 0.839
**Vs. R-ISS stage**	5.9% (-29.7%–43.9%) *p* = 0.697	12.1% (-23.7%–44.2%) *p* = 0.436	3.3% (-34.8%–42.1%) *p* = 0.753	22.7% (-68.5%–89.4%) *p* = 0.545

^a^Range: range of risk threshold to get a positive net benefit in the decision curve analysis (DCA).

Besides, we evaluated the IDI and continuous NRI to test the improvement in the prediction efficiency of the MM-BHAP model (**Table 3**). Compared to ISS stage, the MM-BHAP model showed the statistical improvement of predicting 2-year OS (6.3%, *p* = 0.046, **Figure 2E**) and had a tendency to perform better for the prediction of 6-month and 1-year OS (*p* = 0.088 and 0.080, respectively, **Supplemental Figures S4A, C**), but it was not statistically different for predicting 4-year OS (**Supplemental Figure S4E**) according to the IDI values. Moreover, there was no statistical difference in the prediction of OS between the MM-BHAP model and ISS stage according to the continuous NRI values. Moreover, the prediction efficiency of the MM-BHAP model was comparable to R-ISS stage, with no statistical difference according to the values of IDI and continuous NRI (**Figure 2F** and **Supplemental Figures S4B, D, F**).

In addition, the MM-BHAP model had great calibration for predicting PFS (**Supplemental Figure S5A**). Moreover, it also had globally higher AUC and C-index (**Supplemental Figures S5B, C**), as well as clinical usefulness (**Supplemental Figure S5D**) than that of ISS stage and R-ISS stage in each imputed dataset. Also, there was no statistical difference between the MM-BHAP model and ISS stage/R-ISS stage according to IDI and continuous NRI analyses (**Supplemental Figures S5E, F**).

### Construction of MM-BHAP Stratification

We calculated the total point for each patient according to the nomogram (**Figure 1** and **Table S2**) and divided the patients into low-, medium-, and high-risk subgroups according to the optimal cutoff points calculated by the X-Tile program (32). MM-BHAP stratification stage I was defined as the point ≤110; stage II was defined as the point from 110 to 248; stage III was defined as the point ≥248.

**Figure 3:**
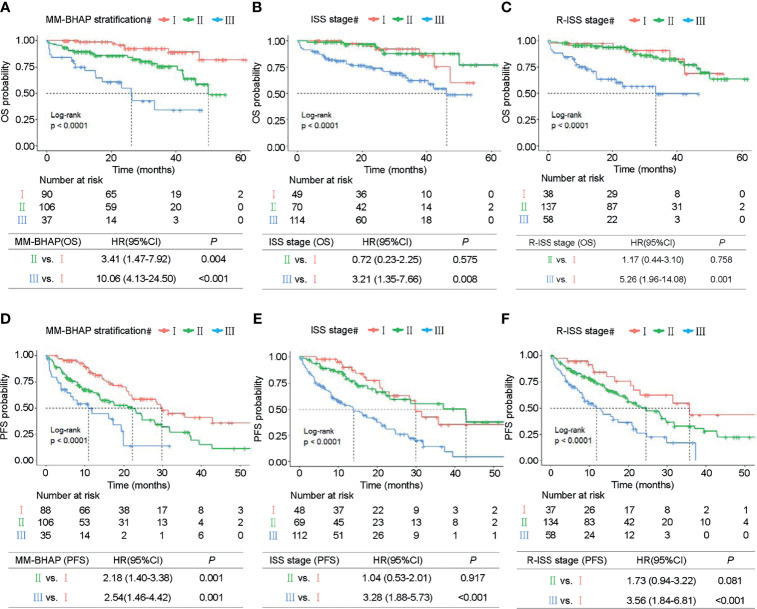
Kaplan–Meier survival curves in the development cohort. OS of MM patients was stratified by the MM-BHAP stratification **(A)**, ISS stage **(B)**, and R-ISS stage **(C)**. PFS was also classified by the three risk stratifications **(D–F)**.

In the development cohort (*N* = 233), the patients were distributed across the three stages of the MM-BHAP stratification as follows (**Table 4** and **Figure 3A**): stage I (38.6%), stage II (45.5%), and stage III (15.9%), with median overall survivals that were not reached (NR), 50.1 months and 26.2 months, respectively. In contrast, both ISS stage (**Figure 3B**) and R-ISS stage (**Figure 3C**) were not satisfactory in stratifying the patients between stages I and II in all the five imputed datasets. Previous studies also indicated the prognostic value of ISS stage II (33) and R-ISS stage II (34) required further improvement.

**Table 4:** Comparison of OS and PFS duration by stage in the development cohort.

Stage	Median OS (months)	Median PFS (months)
**MM-BHAP stratification**		
**I**	NR	29.8
**II**	50.1	22.3
**III**	26.2	10.9
**ISS stage**		
**I**	NR	42.8
**II**	NR	29.9
**III**	46.1	14.0
**R-ISS stage**		
**I**	NR	35.8
**II**	NR	24.5
**III**	33.4	11.9

The median OS of stage III of the MM-BHAP stratification was shorter than that of ISS stage and R-ISS stage (26.2, 46.1, and 33.4 months, respectively), suggesting that the MM-BHAP stratification performed better in identifying a specific group of high-risk patients. We also assessed the stratification for PFS (**Table 4**), indicating that both the MM-BHAP stratification and R-ISS stage had good prognostic stratification (**Figures 3D, F**), while ISS stage was not satisfactory in stratifying patients between stage I and stage II (**Figure 3E**) in all the five imputed datasets.

### Subgroup Analyses

**Figure 4:**
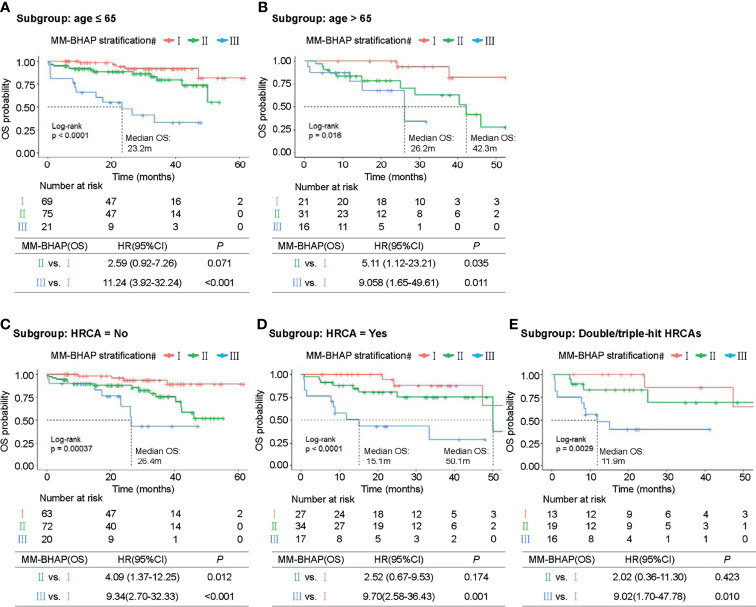
Kaplan–Meier survival curves of specific subgroups stratified by the MM-BHAP stratification in the development cohort. OS curves in the subgroups with different characteristics of age **(A, B)** and HRCA **(C–E)** were shown.

Next, we explored the performance of MM-BHAP stratification in specific groups of patients with age ≤65 or age >65 years and patients with or without HRCA in predicting OS. Both age and HRCA were important prognostic factors, but the MM-BHAP stratification still applied to the four different subgroups (**Figures 4A–D**). Then we further analyzed the applicability of our model in the subgroup of patients with at least two HRCAs recommended by mSMART 3.0, and the result indicated the applicability of MM-BHAP stratification in the double-hit or triple-hit myeloma patients (**Figure 4E**).

**Figure 5:**
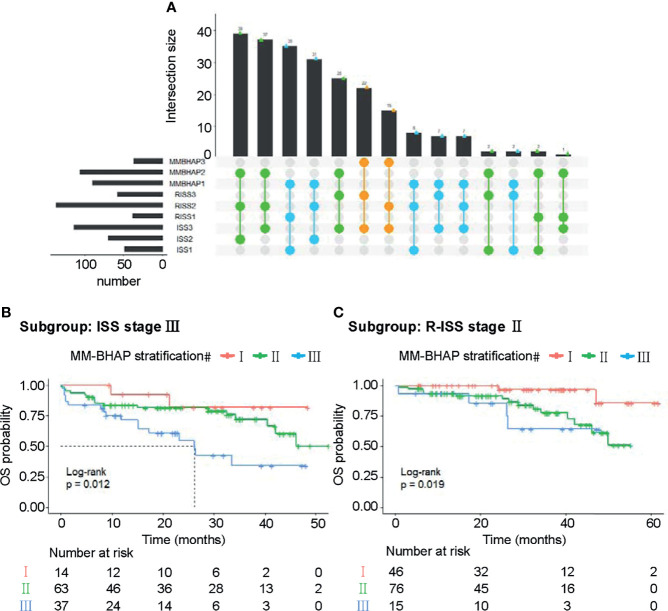
Subgroup analyses about the three risk stratifications in the development cohort. **(A)** The distribution and co-occurrence of the patients respectively classified by the MM-BHAP stratification, R-ISS stage, and ISS stage in the development cohort were displayed. Dots with connected lines represented that the patients coexisted in corresponding different subgroups and the vertical bar graphs reflected the number of these patients. Also, the blue, green, and orange dots respectively represented the co-occurrence of the patients classified by stage I, stage II, and stage II of the MM-BHAP stratification with other subgroups. **(B)** OS curves in the subgroup with ISS stage III stratified by the MM-BHAP model. **(C)** OS curves in the subgroup with R-ISS stage II stratified by the MM-BHAP model.

We also examined the distribution and co-occurrence of the MM-BHAP stratification, R-ISS stage, and ISS stage in the development cohort (*N* = 233) (**Figure 5A**). There were 37 patients among MM-BHAP stage III, all of whom were distributed in ISS stage III simultaneously. Among them, 22 patients existed in R-ISS stage III and 15 patients had R-ISS stage II.

Notably, there was a substantial portion of the patients with R-ISS stage II or ISS stage III, indicating that the two groups of patients needed an accurate stratification. The patients with ISS stage III (**Figure 5B**) could be further divided into three groups by our MM-BHAP stratification. Moreover, in the patients with R-ISS stage II, our model identified a group of patients with favorable outcomes (**Figure 5C**).

### External Validation of the MM-BHAP Model

**Figure 6:**
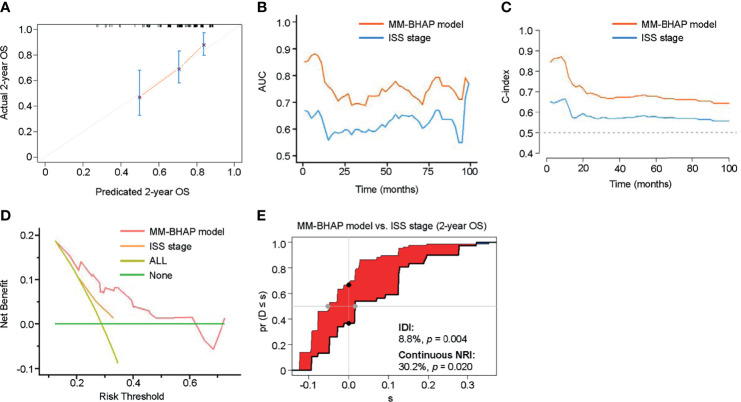
The performance of the MM-BHAP model and ISS stage for predicting OS in the validation cohort. **(A)** The calibration curve of the MM-BHAP model for predicting 2-year OS. **(B)** The time-dependent AUC of the ROC in the two models. **(C)** The time-dependent Harrell’s C-index in the two models. **(D)** The DCA for predicting 2-year OS. **(E)** The IDI and continuous NRI of the MM-BHAP model compared to the ISS stage.

We calculated the total point for each patient of the validation cohort according to the abovementioned nomogram, as a factor for subsequent analyses (35). In the validation cohort, the calibration curve indicated an optimal agreement between the prediction and actual observation for the probability of OS (**Figure 6A** and **Supplemental Figure S6**).

**Table 5** presents other comprehensive evaluations of the MM-BHAP model and ISS stage. Both time-dependent AUC and C-index of the MM-BHAP model were higher than those of the ISS stage, showing a greater prediction performance compared to the ISS stage (**Figures 6B, C**). In the analysis of DCA, the MM-BHAP model had higher net benefits among wider risk thresholds than that of the ISS stage at all time-points (**Figure 6D** and **Supplemental Figure S7**). Surprisingly enough, the MM-BHAP model had a remarkable improvement compared to the ISS stage according to the IDI values (range from 4.8% to 11.8%) and continuous NRI values (range from 30.2% to 56.0%) in predicting OS at different time points (**Figure 6E**, **Supplemental Figure S8** and **Table 5**).

**Table 5:** Comprehensive evaluations of the different models in the validation cohort.

OS	6 months	12 months	24 months
**AUC, n (95% CI)**			
**MM-BHAP**	0.875 (0.816–0.934)	0.797 (0.683–0.912)	0.724 (0.627–0.820)
**ISS stage**	0.650 (0.540–0.761)	0.629 (0.518–0.741)	0.598 (0.503–0.692)
**C-index, n**			
**MM-BHAP**	0.867	0.774	0.692
**ISS stage**	0.649	0.618	0.576
**Range*^a^*, n (%)**			
**MM-BHAP**	2.93%–18.97%	4.70%–23.67%	12.44%–60.88%
**ISS stage**	5.29%–9.24%	8.24%–14.21%	20.15%–33.04%
**AUDC, n**			
**MM-BHAP**	0.0051	0.0087	0.0347
**ISS stage**	0.0008	0.0017	0.0073
**IDI, n (95% CI), *p* value**			
**Vs. ISS stage**	4.8% (1.9%–11.8%) *p* < 0.001	5.4% (1.6%–11.4%) *p* = 0.004	8.8% (2.8%–15.9%) *p* = 0.004
**Continuous NRI, n (95% CI), *p* value**			
**Vs. ISS stage**	56.0% (30.4%–72.7%) *p* < 0.001	46.5% (9.7%–64.0%) *p* = 0.016	30.2% (7.0%–49.1%) *p* = 0.020
**OS**	**48 months**	**60 months**	**72 months**
**AUC, n (95% CI)**			
**MM-BHAP**	0.739 (0.649–0.829)	0.766 (0.675–0.856)	0.735 (0.623–0.847)
**ISS stage**	0.608 (0.512–0.703)	0.646 (0.546–0.748)	0.611 (0.477–0.744)
**C-index, n**			
**MM-BHAP**	0.674	0.677	0.661
**ISS stage**	0.569	0.574	0.565
**Range*^a^*, n (%)**			
**MM-BHAP**	26.62%–95.14%	33.15%–98.04%	38.98%–99.20%
**ISS stage**	38.58%–58.06%	45.87%–66.52%	52.25%–73.23%
**AUDC, n**			
**MM-BHAP**	0.1070	0.1290	0.1202
**ISS stage**	0.0235	0.0308	0.0290
**IDI, n (95% CI), *p* value**			
**Vs. ISS stage**	11.7% (3.6%–18.7%) *p* < 0.001	11.8% (3.3%–18.7%) *p* = 0.008	10.6% (1.9%–16.9%) *p* = 0.012
**Continuous NRI, n (95% CI), *p* value**			
**Vs. ISS stage**	40.6% (6.5%–51.5%) *p* = 0.020	48.0% (6.4%–57.3%) *p* = 0.032	42.5% (0%–57.1%) *p* = 0.052

^a^Range: range of risk threshold to get positive net benefit in the decision curve analysis (DCA) analysis.

Also, the MM-BHAP model had great calibration for predicting PFS (**Supplemental Figure S9A**). At the same time, it performed better in all the analyses of time-dependent AUC and C-index, DCA, IDI, and continuous NRI compared to the ISS stage (**Supplemental Figures S9B–E**).

**Figure 7:**
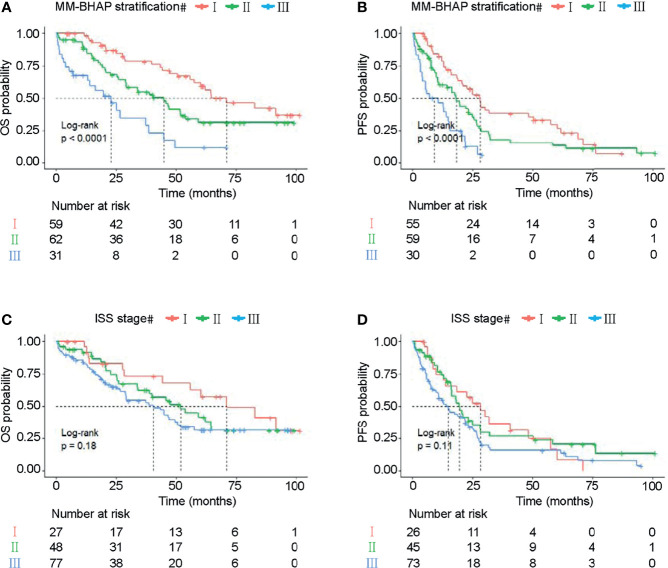
The OS and PFS curves in the validation cohort stratified by the MM-BHAP stratification **(A, B)** and ISS stage **(C, D)**.

The MM-BHAP stratification also categorized patients into three groups well as follows (**Figure 7A**): stage I (38.8%), stage II (40.8%), and stage III (20.4%), with median overall survivals of 71, 44.9, and 22.8 months, respectively. Also, our MM-BHAP stratification had a good stratification for PFS (**Figure 7B**). However, the ISS stage did not perform well in stratifying patients between three stages whether to predict OS (*p* = 0.18) or PFS (*p* = 0.11) (**Figures 7C, D**). Actually, the curves of the ISS stage were superimposable when time was more than approximately 60 months for OS (**Figure 7C**) and when time was more than about 50 months for PFS (**Figure 7D**), which suggested it was not enough for predicting long-term survival to merely utilize the ISS stage.

In the validation cohort, we did not draw comparison of the predictive capacity between the MM-BHAP model and R-ISS stage. This is because the patients were diagnosed between January 2013 and December 2015 in which our center did not completely perform FISH analysis with CD138-purified plasma cells, resulting in the problem taking no unified thresholds to identify HRCA.

### Distribution of Transplant-Eligible Patients

Given that the new stratification included the HCT-CI that was used to assess transplant eligibility, we exploringly analyzed the association between the MM-BHAP stratification at diagnosis with the probability of receiving ASCT afterward. In the whole cohort (*N* = 385), 47 (12.2%) patients underwent ASCT, which were distributed across three stages of the MM-BHAP stratification as follows: stage I (25/149; 16.8%), stage II (18/168; 10.7%), and stage III (4/68; 5.9%). With multiple-comparison analysis by using the Bonferroni method, we found that the proportions between stage I (16.8%) and stage III (5.9%) had statistical difference (*p* < 0.05), suggesting the patients with stage III had less probability to fulfill the eligibility for transplantation in the future compared to patients with stage I.

## Discussion

In this study, we developed and validated a new simple prognostic model (MM-BHAP) integrating comorbidities and tumor burden, for predicting the probability of 6-month, 1-year, 2-year, and 4-year OS of NDMM patients. The novel MM-BHAP model performed well in terms of calibration, discrimination, clinical usefulness, and improvement in prediction, suggesting a good prognostic value for OS and PFS of NDMM patients. Moreover, the performance of the MM-BHAP model was superior to the ISS stage in both development and validation cohorts, while it was partially better at least not worse than the R-ISS stage in the development cohort. Notably, the MM-BHAP stratification categorized patients into three subgroups with clearly different OS or PFS, which was superior to the ISS stage and partially better than the R-ISS stage. Also, it identified a group of high-risk patients with shorter median OS (26.2 months) than that of the ISS stage (46.1 months) and R-ISS stage (33.4 months), indicating an advantage of defining truly high-risk patients in the real-world population. Furthermore, it enhanced the differential power of the ISS stage and R-ISS stage, with reclassifications in patients with ISS stage III or R-ISS stage II.

The MM-BHAP model was composed of four widely accessible factors of β2-MG, HCT-CI, ALB, and PBPC. β2-MG and ALB were typically prognostic factors integrated into the ISS stage (5). In contrast, we divided the value of ALB into three levels (≤24.5; >24.5 and ≤35; >35 g/l), based on the restricted cubic splines. With regard to PBPC, its importance was second only to HCT-CI in the MM-BHAP model. Multiple studies demonstrated that high levels of circulating plasma cells (CPCs) in MM patients were associated with worse prognosis (36–40) and even the prognosis of the MM patients with ≥5% CPCs was equivalent to that of the patients with plasma cell leukemia (41). In addition, it was evidenced that HCT-CI was independently associated with poor OS of MM patients undergoing HSCT (26, 27). It was of concern that HCT-CI also had potential to predict outcomes of the patients with newly diagnosed hematologic malignancies, not merely the patients after HSCT. A multicenter study firstly developed and validated a prognostic model incorporating HCT-CI at diagnosis, to estimate risks of mortality in acute myeloid leukemia (42). Nevertheless, little is known about the prognostic impact of HCT-CI at the time of diagnosis on the outcomes of NDMM patients. Herein, we systematically examined the prognostic value of HCT-CI at diagnosis which contributed the most to our MM-BHAP model. To our knowledge, the present study is the first to develop and validate a novel prognostic model integrating comorbidities measured by HCT-CI and tumor burden reflected by β2-MG, ALB, and PBPC.

Although HRCA was not selected into our model, it had robust prognostic implications. However, the application of novel agents and ASCT seemed to improve the poor outcomes of the patients with certain HRCAs. For instance, the adverse impact of t(4;14) could be partly overcome by bortezomib (43) and the inferior outcome of del(17p) could be improved by maintenance therapy and ASCT especially in transplant eligible patients (44, 45), suggesting highly heterogeneous outcomes of the patients with HRCA. Therefore, there was an urgent need to further identify the ultra-high-risk subgroup within the patients harboring HRCA. In our cohort, the overall survival of the patients with HRCA or double/triple-hit HRCA could be further differentiated by our MM-BHAP stratification, maybe partially because of diverse therapy regimens and treatment intensity resulting from the burden of different comorbidities.

It was noteworthy that the R-ISS stage was inclined to present a better prognostic stratification for PFS compared to OS. As we all know, the R-ISS stage was completely composed of disease-related factors, which mainly reflected the inherent biological characteristics of myeloma (46–49). However, the overall survival of MM was dependent on not only the disease progression but also the host features such as comorbidities, performance status, treatment intention, and socioeconomic support, which were more likely to affect the drug accessibility and treatment integrity. Moreover, the R-ISS stage was developed in the highly selected cohorts within clinical trials (6) in which the patients with comorbidities were not underrepresented. Therefore, the R-ISS stage might be more applicable to the patients defined as “fit,” and it was essential to combine disease-related and patient-related prognostic factors in predicting overall survival in the real-world unselected population.

It was well known that the intermediate-risk group of the R-ISS stage was an exclusionary definition, which was an indication of the high heterogeneity of genetic background and outcomes. Further subdivision may redefine the outcomes of some patients, thereby achieving precise and personalized management. In our study, about 60% of the patients were among R-ISS stage II, which was similar to a previous R-ISS study (62%) (6). Moreover, among these patients, our model could identify a subgroup of patients with favorable outcomes. Furthermore, the MM-BHAP stratification could distinguish a group of patients with higher risk in the subgroup with ISS stage III, which further verified its applicability in some specific subsets.

Yet, there were still some limitations in our study. This study only included patients from a single center and needed further confirmations from larger multicenter cohorts. Moreover, this is a real-world retrospective cohort study, showing some missing data inevitably, but we handled missing data by using MICE to minimize the impact of data deficiency. HRCA was a known prognostic factor, and our subgroup analysis also suggested that patients with HRCA had poor median OS. However, it was finally not selected into our model, maybe because the impact was undervalued due to data deficiency, or it was knocked out since other variables contribute more in LASSO analysis. Besides, the comparison between MM-BHAP model and R-ISS stage was established only in the development cohort, requiring further validation. However, the effect of missing value on our model was minor because the finally selected variables included in the nomogram were all complete data in both development cohort and validation cohort. Moreover, the MM-BHAP stratification could definitely categorize patients into different groups with distinct outcomes.

In conclusion, our study was the first to use HCT-CI at diagnosis to predict the outcome of NDMM patients. Utilizing widely available prognostic factors, we constructed a novel simple MM-BHAP stratification combining tumor burden with comorbidities for better differentiation of real-world unselected patients with NDMM. It was expected to have a more accurate prediction for outcome uniting the MM-BHAP stratification and the current risk stratifications.

## Data Availability Statement

The original contributions presented in the study are included in the article/**Supplementary Material**. Further inquiries can be directed to the corresponding authors.

## Ethics Statement

The study was reviewed and approved by the Ethics Committee of Xijing Hospital of Air Force Medical University.

## Author Contributions

GG, HT, SJ, and LB designed and conducted the research. JF, LX, TZ, HG, LY, QB, and RL enrolled the patients. SJ, LB, YC, XL, BT, and YG collected the data. SJ and HT analyzed the data and wrote the paper. GG and HT reviewed the manuscript. All authors contributed to the article and approved the submitted version.

## Funding

This study was supported by the National Natural Science Foundation of China (No. 81970190 for GG; No. 82100218 for HT; No. 81900207 for LX), the Translation Research Grant of NCRCH (No. 2020ZKMC01 for GG), and the Key Research and Development Program in Shaanxi Province (No. 2019ZDLSF02-02 for GG).

## Conflict of Interest

The authors declare that the research was conducted in the absence of any commercial or financial relationships that could be construed as a potential conflict of interest.

## Publisher’s Note

All claims expressed in this article are solely those of the authors and do not necessarily represent those of their affiliated organizations, or those of the publisher, the editors and the reviewers. Any product that may be evaluated in this article, or claim that may be made by its manufacturer, is not guaranteed or endorsed by the publisher.

## References

[1] Siegel RL Miller KD Jemal A. Cancer Statistics, 2016. CA Cancer J Clin (2016) 66:7–30. doi: 10.3322/caac.21332 26742998

[2] Perrot A Lauwers-Cances V Tournay E Hulin C Chretien ML Royer B. Development and Validation of a Cytogenetic Prognostic Index Predicting Survival in Multiple Myeloma. J Clin Oncol (2019) 37:1657–65. doi: 10.1200/JCO.18.00776 PMC6804890 31091136

[3] Bringhen S Offidani M Palmieri S Pisani F Rizzi R Spada S. Early Mortality in Myeloma Patients Treated With First-Generation Novel Agents Thalidomide, Lenalidomide, Bortezomib at Diagnosis: A Pooled Analysis. Crit Rev Oncol Hematol (2018) 130:27–35. doi: 10.1016/j.critrevonc.2018.07.003 30196909

[4] Ríos-Tamayo R Sáinz J Martínez-López J Puerta JM Chang DY Rodríguez T. Early Mortality in Multiple Myeloma: The Time-Dependent Impact of Comorbidity: A Population-Based Study in 621 Real-Life Patients. Am J Hematol (2016) 91:700–4. doi: 10.1002/ajh.24389 27074204

[5] Greipp PR San MJ Durie BG Crowley JJ Barlogie B Bladé J. International Staging System for Multiple Myeloma. J Clin Oncol (2005) 23:3412–20. doi: 10.1200/JCO.2005.04.242 15809451

[6] Palumbo A Avet-Loiseau H Oliva S Lokhorst HM Goldschmidt H Rosinol L. Revised International Staging System for Multiple Myeloma: A Report From International Myeloma Working Group. J Clin Oncol (2015) 33:2863–9. doi: 10.1200/JCO.2015.61.2267 PMC4846284 26240224

[7] Kuiper R van Duin M van Vliet MH Broijl A van der Holt B El JL. Prediction of High- and Low-Risk Multiple Myeloma Based on Gene Expression and the International Staging System. Blood (2015) 126:1996–2004. doi: 10.1182/blood-2015-05-644039 26330243 PMC4616233

[8] Sun C Li H Mills RE Guan Y. Prognostic Model for Multiple Myeloma Progression Integrating Gene Expression and Clinical Features. Gigascience (2019) 8(12):giz153. doi: 10.1093/gigascience/giz153 31886876 PMC6936209

[9] Wang W Xu SW Zhu XY Guo QY Zhu M Mao XL. Identification and Validation of a Novel RNA-Binding Protein-Related Gene-Based Prognostic Model for Multiple Myeloma. Front Genet (2021) 12:665173. doi: 10.3389/fgene.2021.665173 33981333 PMC8107400

[10] Mosquera OA González PM Díaz AJ Antelo RB Alonso VN Bendaña LA. Survival Prediction and Treatment Optimization of Multiple Myeloma Patients Using Machine-Learning Models Based on Clinical and Gene Expression Data. Leukemia (2021) 35:2924–35. doi: 10.1038/s41375-021-01286-2 34007046

[11] Kuiper R Broyl A de Knegt Y van Vliet MH van Beers EH van der Holt B. A Gene Expression Signature for High-Risk Multiple Myeloma. Leukemia (2012) 26:2406–13. doi: 10.1038/leu.2012.127 22722715

[12] Shaughnessy JJ Zhan F Burington BE Huang Y Colla S Hanamura I. A Validated Gene Expression Model of High-Risk Multiple Myeloma is Defined by Deregulated Expression of Genes Mapping to Chromosome 1. Blood (2007) 109:2276–84. doi: 10.1182/blood-2006-07-038430 17105813

[13] Decaux O Lodé L Magrangeas F Charbonnel C Gouraud W Jézéquel P. Prediction of Survival in Multiple Myeloma Based on Gene Expression Profiles Reveals Cell Cycle and Chromosomal Instability Signatures in High-Risk Patients and Hyperdiploid Signatures in Low-Risk Patients: A Study of the Intergroupe Francophone Du Myélome. J Clin Oncol (2008) 26:4798–805. doi: 10.1200/JCO.2007.13.8545 18591550

[14] Huang HY Wang Y Wang WD Wei XL Gale RP Li JY. A Prognostic Survival Model Based on Metabolism-Related Gene Expression in Plasma Cell Myeloma. Leukemia (2021) 35:3212–22. doi: 10.1038/s41375-021-01206-4 33686197

[15] Zhou M Zhao H Wang Z Cheng L Yang L Shi H. Identification and Validation of Potential Prognostic lncRNA Biomarkers for Predicting Survival in Patients With Multiple Myeloma. J Exp Clin Cancer Res (2015) 34:102. doi: 10.1186/s13046-015-0219-5 26362431 PMC4567800

[16] Cheng Q Cai L Zhang Y Chen L Hu Y Sun C. Circulating Plasma Cells as a Biomarker to Predict Newly Diagnosed Multiple Myeloma Prognosis: Developing Nomogram Prognostic Models. Front Oncol (2021) 11:639528. doi: 10.3389/fonc.2021.639528 33747963 PMC7973368

[17] Al Saleh AS Sidiqi MH Dispenzieri A Kapoor P Muchtar E. Hematopoietic Score Predicts Outcomes in Newly Diagnosed Multiple Myeloma Patients. Am J Hematol (2020) 95:4–9. doi: 10.1002/ajh.25657. 31612526 PMC7377299

[18] Mellors PW Binder M Ketterling RP Greipp PT Baughn LB Peterson JF. Metaphase Cytogenetics and Plasma Cell Proliferation Index for Risk Stratification in Newly Diagnosed Multiple Myeloma. Blood Adv (2020) 4:2236–44. doi: 10.1182/bloodadvances.2019001275 PMC7252538 32442300

[19] Cook G Royle KL Pawlyn C Hockaday A Shah V Kaiser MF. A Clinical Prediction Model for Outcome and Therapy Delivery in Transplant-Ineligible Patients With Myeloma (UK Myeloma Research Alliance Risk Profile): A Development and Validation Study. Lancet Haematol (2019) 6:e154–66. doi: 10.1016/S2352-3026(18)30220-5 PMC6391517 30738834

[20] Terebelo HR Abonour R Gasparetto CJ Toomey K Durie B Hardin JW. Development of a Prognostic Model for Overall Survival in Multiple Myeloma Using the Connect® MM Patient Registry. Br J Haematol (2019) 187:602–14. doi: 10.1111/bjh.16139 PMC6899784 31382320

[21] Hou ZL Kang Y Yang GZ Wang Z Wang F Yu YX. Pleural Effusion-Based Nomogram to Predict Outcomes in Unselected Patients With Multiple Myeloma: A Large Single Center Experience. Ann Hematol (2021) 100:1789–801. doi: 10.1007/s00277-021-04484-1 33715037

[22] Sorror ML Maris MB Storb R Baron F Sandmaier BM Maloney DG. Hematopoietic Cell Transplantation (HCT)-Specific Comorbidity Index: A New Tool for Risk Assessment Before Allogeneic HCT. Blood (2005) 106:2912–9. doi: 10.1182/blood-2005-05-2004 PMC1895304 15994282

[23] Djebbari F Panitsas F Eyre TA Prodger C Davies F Burton K. Infection-Related Morbidity in a Large Study of Transplant non-Eligible Newly Diagnosed Myeloma Patients Treated With UK Standard of Care. Haematologica (2020) 105:e474–9. doi: 10.3324/haematol.2019.240762 PMC7556501 33054067

[24] Sorror ML Logan BR Zhu X Rizzo JD Cooke KR McCarthy PL. Prospective Validation of the Predictive Power of the Hematopoietic Cell Transplantation Comorbidity Index: A Center for International Blood and Marrow Transplant Research Study. Biol Blood Marrow Transplant (2015) 21:1479–87. doi: 10.1016/j.bbmt.2015.04.004 PMC4512746 25862591

[25] Thakar MS Broglie L Logan B Artz A Bunin N Burroughs LM. The Hematopoietic Cell Transplant Comorbidity Index Predicts Survival After Allogeneic Transplant for Nonmalignant Diseases. Blood (2019) 133:754–62. doi: 10.1182/blood-2018-09-876284 PMC6376282 30545834

[26] Saad A Mahindra A Zhang MJ Zhong X Costa LJ Dispenzieri A. Hematopoietic Cell Transplant Comorbidity Index is Predictive of Survival After Autologous Hematopoietic Cell Transplantation in Multiple Myeloma. Biol Blood Marrow Transplant (2014) 20:402–8. doi: 10.1016/j.bbmt.2013.12.557 PMC3961011 24342394

[27] Obiozor C Subramaniam DP Divine C Shune L Singh AK Lin TL. Evaluation of Performance Status and Hematopoietic Cell Transplantation Specific Comorbidity Index on Unplanned Admission Rates in Patients With Multiple Myeloma Undergoing Outpatient Autologous Stem Cell Transplantation. Biol Blood Marrow Transplant (2017) 23:1641–5. doi: 10.1016/j.bbmt.2017.06.001 28603071

[28] Rajkumar SV Dimopoulos MA Palumbo A Blade J Merlini G Mateos MV. International Myeloma Working Group Updated Criteria for the Diagnosis of Multiple Myeloma. Lancet Oncol (2014) 15:e538–48. doi: 10.1016/S1470-2045(14)70442-5 25439696

[29] Ross FM Avet-Loiseau H Ameye G Gutiérrez NC Liebisch P O Connor S. Report From the European Myeloma Network on Interphase FISH in Multiple Myeloma and Related Disorders. Haematologica (2012) 97:1272–7. doi: 10.3324/haematol.2011.056176 PMC3409827 22371180

[30] Friedman J Hastie T Tibshirani R. Regularization Paths for Generalized Linear Models *via* Coordinate Descent. J Stat Software (2010) 33:1–22. doi: 10.18637/jss.v033.i01 PMC2929880 20808728

[31] Fitzgerald M Saville BR Lewis RJ. Decision Curve Analysis. JAMA (2015) 313:409–10. doi: 10.1001/jama.2015.37 25626037

[32] Camp RL Dolled-Filhart M Rimm DL. X-Tile: A New Bio-Informatics Tool for Biomarker Assessment and Outcome-Based Cut-Point Optimization. Clin Cancer Res (2004) 10:7252–9. doi: 10.1158/1078-0432.CCR-04-0713 15534099

[33] Cho H Yoon DH Lee JB Kim SY Moon JH Do YR. Comprehensive Evaluation of the Revised International Staging System in Multiple Myeloma Patients Treated With Novel Agents as a Primary Therapy. Am J Hematol (2017) 92:1280–6. doi: 10.1002/ajh.24891 28833417

[34] Galieni P Travaglini F Vagnoni D Ruggieri M Caraffa P Bigazzi C. The Detection of Circulating Plasma Cells may Improve the Revised International Staging System (R-ISS) Risk Stratification of Patients With Newly Diagnosed Multiple Myeloma. Br J Haematol (2021) 193:542–50. doi: 10.1111/bjh.17118 33792026

[35] Wang Y Li J Xia Y Gong R Wang K Yan Z. Prognostic Nomogram for Intrahepatic Cholangiocarcinoma After Partial Hepatectomy. J Clin Oncol (2013) 31:1188–95. doi: 10.1200/JCO.2012.41.5984 23358969

[36] Nowakowski GS Witzig TE Dingli D Tracz MJ Gertz MA Lacy MQ. Circulating Plasma Cells Detected by Flow Cytometry as a Predictor of Survival in 302 Patients With Newly Diagnosed Multiple Myeloma. Blood (2005) 106:2276–9. doi: 10.1182/blood-2005-05-1858 PMC1895270 15961515

[37] Vagnoni D Travaglini F Pezzoni V Ruggieri M Bigazzi C Dalsass A. Circulating Plasma Cells in Newly Diagnosed Symptomatic Multiple Myeloma as a Possible Prognostic Marker for Patients With Standard-Risk Cytogenetics. Br J Haematol (2015) 170:523–31. doi: 10.1111/bjh.13484 26010293

[38] Gonsalves WI Rajkumar SV Dispenzieri A Dingli D Timm MM Morice WG. Quantification of Circulating Clonal Plasma Cells *via* Multiparametric Flow Cytometry Identifies Patients With Smoldering Multiple Myeloma at High Risk of Progression. Leukemia (2017) 31:130–5. doi: 10.1038/leu.2016.205 PMC5244483 27457702

[39] Chakraborty R Muchtar E Kumar SK Jevremovic D Buadi FK Dingli D. Serial Measurements of Circulating Plasma Cells Before and After Induction Therapy Have an Independent Prognostic Impact in Patients With Multiple Myeloma Undergoing Upfront Autologous Transplantation. Haematologica (2017) 102:1439–45. doi: 10.3324/haematol.2017.166629 PMC5541877 28473618

[40] Bianchi G Kyle RA Larson DR Witzig TE Kumar S Dispenzieri A. High Levels of Peripheral Blood Circulating Plasma Cells as a Specific Risk Factor for Progression of Smoldering Multiple Myeloma. Leukemia (2013) 27:680–5. doi: 10.1038/leu.2012.237 PMC3597230 22902364

[41] Ravi P Kumar SK Roeker L Gonsalves W Buadi F Lacy MQ. Revised Diagnostic Criteria for Plasma Cell Leukemia: Results of a Mayo Clinic Study With Comparison of Outcomes to Multiple Myeloma. Blood Cancer J (2018) 8:116. doi: 10.1038/s41408-018-0140-1 30442928 PMC6238010

[42] Sorror ML Storer BE Fathi AT Gerds AT Medeiros BC Shami P. Development and Validation of a Novel Acute Myeloid Leukemia-Composite Model to Estimate Risks of Mortality. JAMA Oncol (2017) 3:1675–82. doi: 10.1001/jamaoncol.2017.2714 PMC5824273 28880971

[43] Avet-Loiseau H Leleu X Roussel M Moreau P Guerin-Charbonnel C Caillot D. Bortezomib Plus Dexamethasone Induction Improves Outcome of Patients With T(4;14) Myeloma But Not Outcome of Patients With Del(17p). J Clin Oncol (2010) 28:4630–4. doi: 10.1200/JCO.2010.28.3945 20644101

[44] Neben K Lokhorst HM Jauch A Bertsch U Hielscher T van der Holt B. Administration of Bortezomib Before and After Autologous Stem Cell Transplantation Improves Outcome in Multiple Myeloma Patients With Deletion 17p. Blood (2012) 119:940–8. doi: 10.1182/blood-2011-09-379164 22160383

[45] Merz M Hielscher T Seckinger A Hose D Mai EK Raab MS. Baseline Characteristics, Chromosomal Alterations, and Treatment Affecting Prognosis of Deletion 17p in Newly Diagnosed Myeloma. Am J Hematol (2016) 91:E473–7. doi: 10.1002/ajh.24533 27508939

[46] Fonseca R Bergsagel PL Drach J Shaughnessy J Gutierrez N Stewart AK. International Myeloma Working Group Molecular Classification of Multiple Myeloma: Spotlight Review. Leukemia (2009) 23:2210–21. doi: 10.1038/leu.2009.174 PMC2964268 19798094

[47] Cassuto JP Krebs BP Viot G Dujardin P Masseyeff R. Beta 2 Microglobulin, a Tumour Marker of Lymphoproliferative Disorders. Lancet (1978) 2:108–9. doi: 10.1016/s0140-6736(78)91428-9 78278

[48] Bataille R Durie BG Grenier J Sany J. Prognostic Factors and Staging in Multiple Myeloma: A Reappraisal. J Clin Oncol (1986) 4:80–7. doi: 10.1200/JCO.1986.4.1.80 3510284

[49] Dimopoulos MA Barlogie B Smith TL Alexanian R. High Serum Lactate Dehydrogenase Level as a Marker for Drug Resistance and Short Survival in Multiple Myeloma. Ann Intern Med (1991) 115:931–5. doi: 10.7326/0003-4819-115-12-931 1952489

