# Supplementary material for: Development and Validation of a Novel Prognostic Model for Overall Survival in Newly Diagnosed Multiple Myeloma Integrating Tumor Burden and Comorbidities
Source: Front Oncol. 2022 Mar 17;12:805702. doi: 10.3389/fonc.2022.805702 (PMC8968003; doi:10.3389/fonc.2022.805702)
Supplement: Supplementary file 1 [file DataSheet_1.pdf]

Supplementary Material

Methods

1.Variable selection

There were 13 variables with *P* value < 0.10, except for ISS stage and R-ISS stage, in the univariate Cox regression analysis (Table 1): age, HCT-CI, WBC, NEU, LYM, MONO, HGB, β2-MG, ALB, serum calcium, HRCA, PBPC and ASCT. We subjected them to the LASSO analysis, and five variables of β2-MG, HCT-CI, ALB, PBPC and MONO were steadily screened out in each imputed dataset (Figure S2A, B).

The HR of MONO (>0.9 vs. ≤0.9 $\times 10^9$/L) was 4.25 (95%CI: 1.68-10.75), but there were just 8 (3.4%) patients with MONO >0.9 $\times 10^9$/L in the development cohort (Table S1). In order to evaluate its contribution to prediction, we developed two models. One of them consisted of five variables including MONO while the other did not. Subsequently, we compared their discrimination by the time-dependent AUC and time-dependent C-index analyses, turning out to be similar between the two models (Figure S2C, D). Considering simplicity, we determined to select β2-MG, HCT-CI, ALB and PBPC to develop the prognostic model.

2.The R packages involved in the study

The R package 'lattice', 'MASS', 'nnet', 'foreign' and 'mice' were used for MICE. The R package 'rms', 'survminer' and 'ggplot2' were used for restricted cubic splines based on cox regression. The R package 'survival' and 'glmnet' were used for LASSO analysis. The R package 'survival' and 'plyr' were used for cox proportional hazards model. The R package 'rms' was used for nomogram. The R package 'rms' and 'nomogramFormula' were used to calculated point for each patient based on the nomogram. The R package 'rms' was used for calibration curve. The R package 'survival', 'riskRegression', 'ggplot2' and 'ggprism' were used for comparing the time-dependent AUC. The R package 'rms' and 'pec' were used for comparing the time-dependent C-index. The R package 'rms', 'ggDCA', 'survival', 'ggplot' and 'ggprism' were used for DCA. The R package 'survival', 'survIDINRI' and 'survC1' were used to calculate IDI and continuous NRI. The R package 'survminer' and 'survival' were used for survival curves.

Table S1. Baseline characteristics of development and validation cohorts

Characteristics	Development (*N*=233)	Validation (*N*=152)
**Patient specific**		
**Age (years), n (%)**		
**≤65**	165(70.8)	118(77.6)
**>65**	68(29.2)	34(22.4)
**Sex, n (%)**		
**female**	87(37.3)	65(42.8)
**Male**	146(62.7)	87(57.2)
**BMI (kg/m^2), n (%)**		
**≤22.5**	103(44.2)	56(36.8)
**＞22.5&≤25.5**	82(35.2)	55(36.2)
**＞25.5**	44(18.9)	27(17.8)
**missing**	4(1.7)	14(9.2)
**History of hypertention, n (%)**		
**No**	178(76.4)	107(70.4)
**Yes**	55(23.6)	45(29.6)
**History of thrombosis, n (%)**		
**No**	212(91.0)	141(92.8)

**Yes**	21(9.0)	11(7.2)
**HCT-CI (points), n (%)**		
**≤1**	154(66.1)	93(61.2)
**>1**	79(33.9)	59(38.8)
**Disease specific**		
**WBC (×10^9/L), n (%)**		
**≤8.85**	214(91.8)	139(91.4)
**>8.85**	19(8.2)	13(8.6)
**Neu (×10^9/L), median**	2.82	2.98
**LYM (×10^9/L), median**	1.47	1.41
**MONO (×10^9/L), n (%)**		
**≤0.9**	225(96.6)	141(92.8)
**>0.9**	8(3.4)	11(7.2)
**HGB (g/L), n (%)**		
**≤70**	39(16.7)	25(16.4)
**>70&≤120**	147(63.1)	100(65.8)
**>120**	47(20.2)	27(17.8)
**PLT (×10^9/L), n (%)**		
**≤228**	191(82)	128(84.2)
**>228**	42(18)	24(15.8)
**β2-MG (mg/L), n (%)**		

**<3.5**	60(25.8)	34(22.4)
**≥3.5&<5.5**	63(27.0)	41(27.0)
**≥5.5**	110(47.2)	77(50.7)
**ALB (g/L), n (%)**		
**≤24.5**	21(9.0)	11(7.2)
**>24.5&≤35**	95(40.8)	56(36.8)
**>35**	117(50.2)	85(55.9)
**Ca^{2+} (mmol/L), median**	2.26	2.20
**LDH (IU/L), n (%)**		
**≤300**	182(78.1)	95(62.5)
**>300**	17(7.3)	11(7.2)
**missing**	34(14.6)	46(30.3)
**HRCA, n (%)**		
**No**	128(54.9)	-
**Yes**	35(15.0)	-
**missing**	70(30.0)	-
**BMPC (%), n (%)**		
**≤61.7**	189(81.1)	125(82.2)
**>61.7**	44(18.9)	27(17.8)
**PBPC (%), n (%)**		

**0**	186(79.8)	125(82.2)
**>0&<2.7**	24(10.3)	19(12.5)
**≥2.7**	23(9.9)	8(5.3)
**DS stage, n (%)**		
**I**	20(8.6)	8(5.3)
**II**	32(13.7)	25(16.4)
**III**	180(77.3)	119(78.3)
**ISS stage, n (%)**		
**I**	49(21.0)	27(17.8)
**II**	70(30.0)	48(31.6)
**III**	114(48.9)	77(51.7)
**R-ISS stage, n (%)**		
**I**	22(9.4)	-
**II**	117(50.2)	-
**III**	32(13.7)	-
**missing**	62(26.6)	-
**Novel therapy**		
**PIs or IMiDs, n (%)**	229(98.3)	149(98.0)
**Other, n (%)**	4(1.7)	3(2.0)
**ASCT, n (%)**		
**No**	207(88.8)	131(86.2)

**Yes**	26(11.2)	21(13.8)

Abbreviations: BMI: body mass index; HCT-CI: hematopoietic cell transplantation-comorbidity index; WBC: white blood cell; Neu: neutrophil granulocyte; LYM: lymphocyte; MONO: monocyte; HGB: hemoglobin; PLT: platelet; β2-MG: β2-microglobulin; ALB: albumin; LDH: lactate dehydrogenase; HRCA: high-risk chromosomal abnormalities (t (4;14) and /or t (14;16) and/or del(17p)); BMPC: bone marrow plasma cells; PBPC: peripheral blood plasma cells; DS: Durie/ Salmon staging system; ISS: International Staging System; R-ISS: Revised International Staging System; PIs: proteasome inhibitors; IMiDs: immunomodulatory drugs; ASCT: autologous stem-cell transplantation.

Table S2. The specific point of each variable in the nomogram

Variable	point
**Albumin (g/L)**	
**≤24.5**	93
**>24.5&≤35**	59
**>35**	0
**β2-MG (mg/L)**	
**<3.5**	0
**≥3.5&<5.5**	60
**≥5.5**	92
**PBPC (%)**	
**0**	0
**>0&<2.7**	72
**≥2.7**	91
**HCT-CI (points)**	
**≤1**	0
**>1**	100

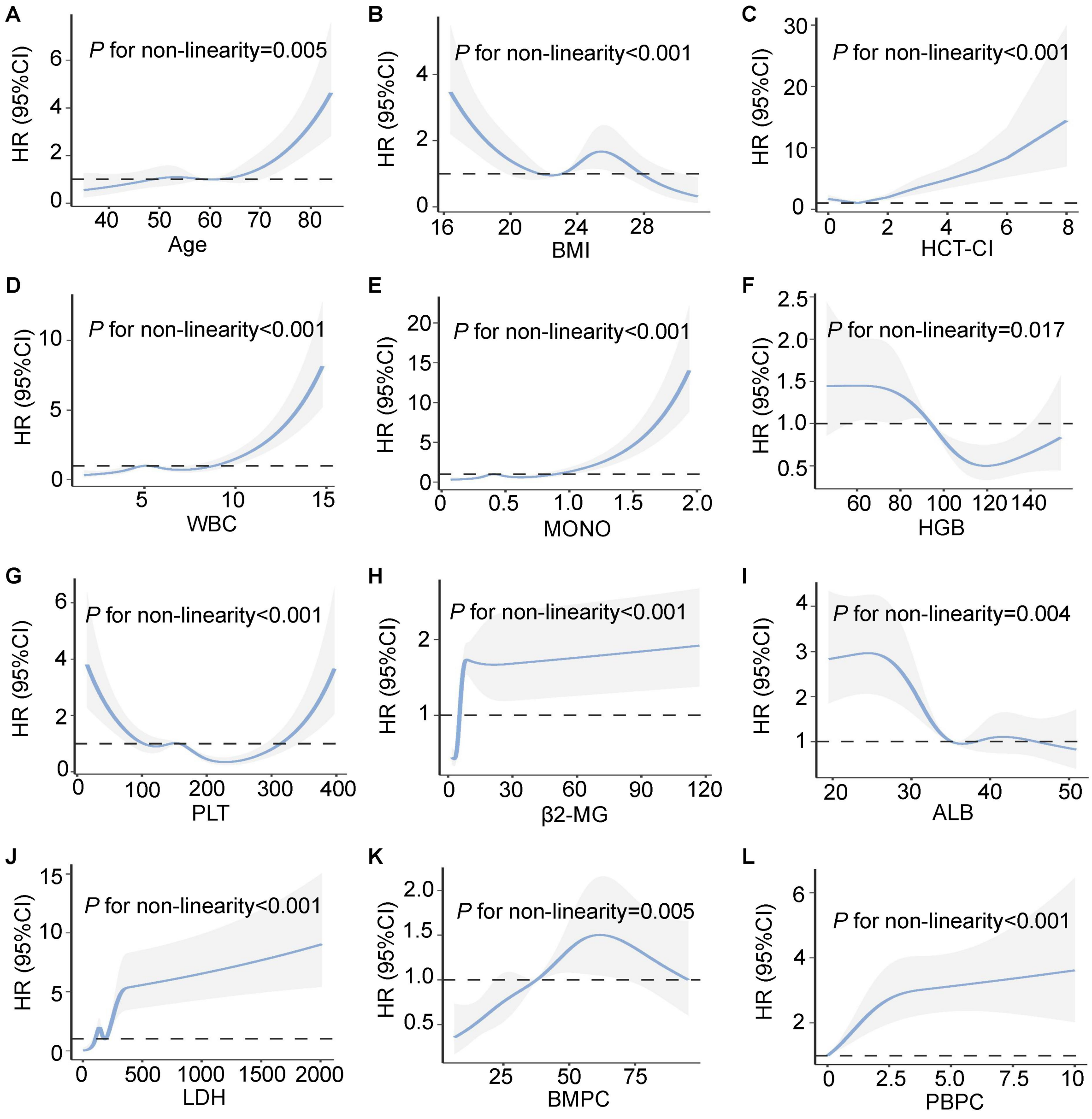

Figure S1. The nonlinear association between different continuous variables with OS examined by restricted cubic splines based on cox regression. The variable was transformed into categorical variable according to the cutoff points when the *P* value for non-linearity < 0.05.

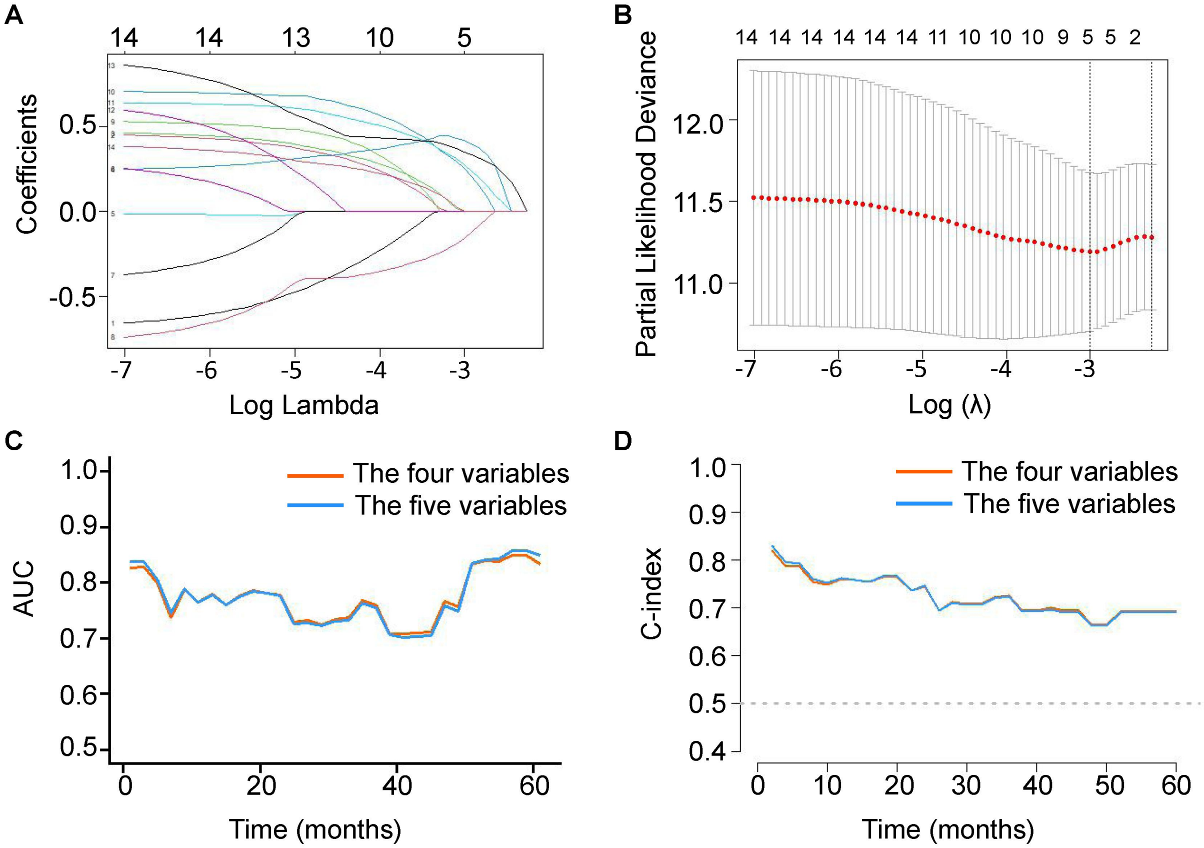

Figure S2. Variable selection. (A) Coefficients of each variable in the LASSO analysis. (B) 1000 bootstrap resamples by LASSO analysis. (C) The time-dependent AUC of the ROC in the two models including four or five variables. (D) The time-dependent Harrell's C-index in the two models containing four or five variables.

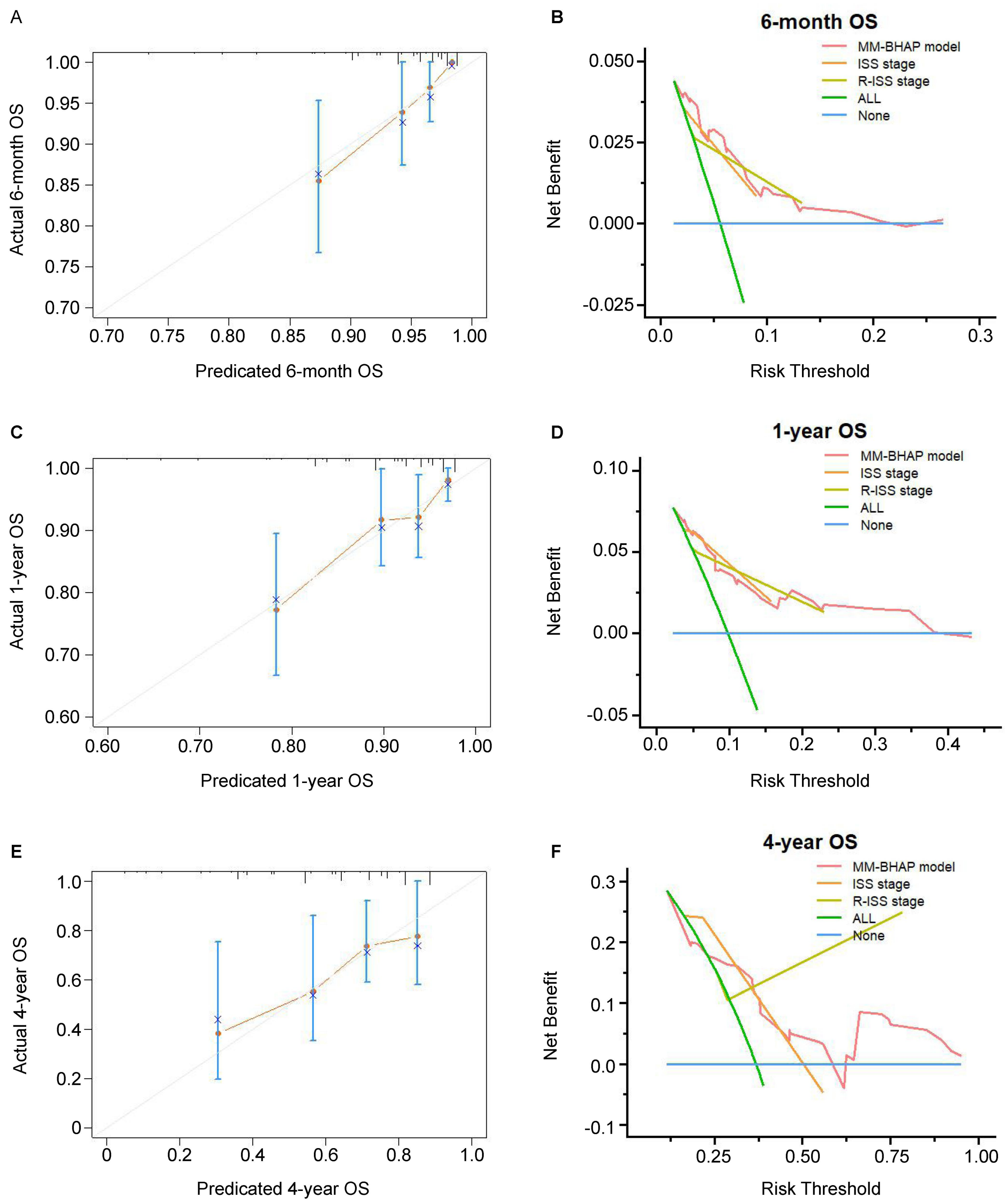

Figure S3. The calibration curve (A, C, E) of the MM-BHAP model and DCA (B, D, F) of the MM-BHAP model, ISS stage and R-ISS stage for predicting 6-month, 1-year and 4-year OS in the development cohort.

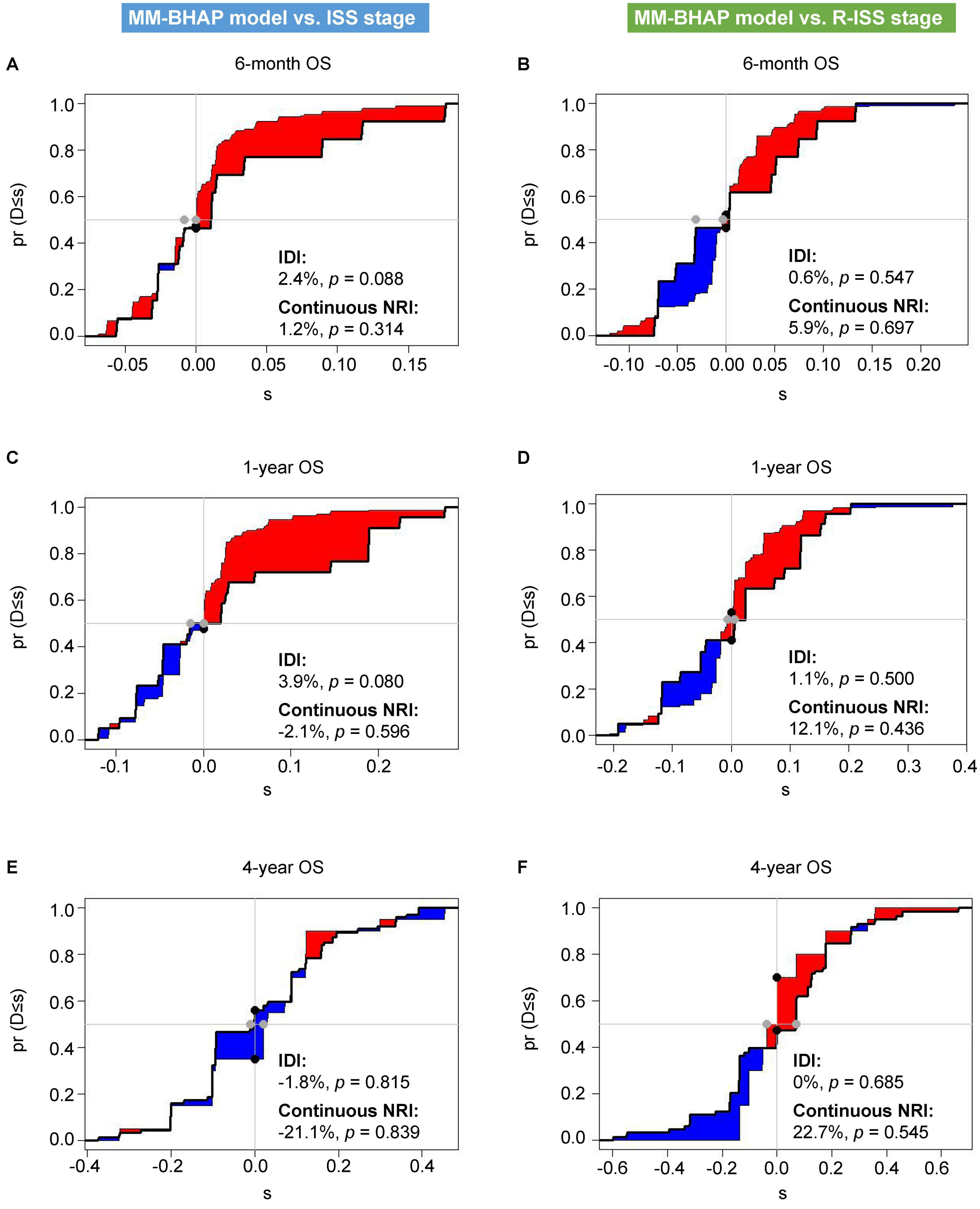

Figure S4. The improvement in prediction of the MM-BHAP model compared to ISS stage (A, C, E) or R-ISS stage (B, D, F) according to IDI and continuous NRI in the development cohort.

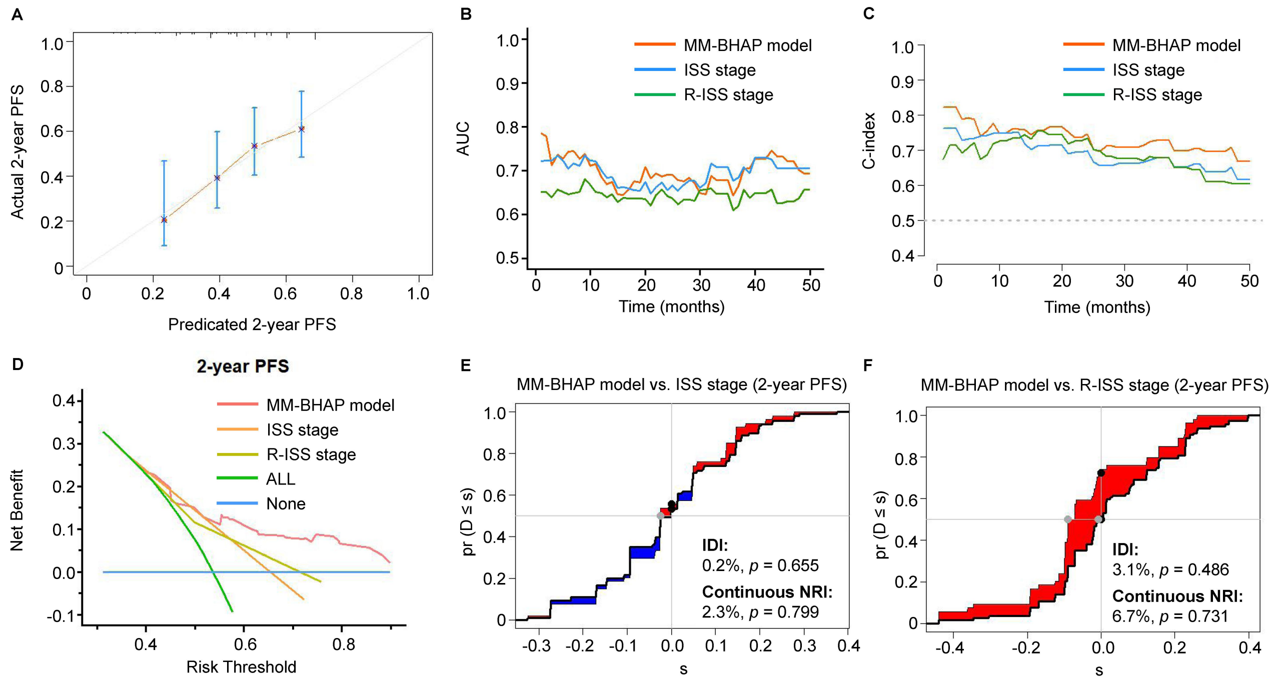

Figure S5. The performance of the MM-BHAP model, ISS stage and R-ISS stage for predicting PFS in the development cohort. (A) The calibration curve of the MM-BHAP model for predicting 2-year PFS. (B) The time-dependent AUC of the ROC in the three models. (C) The time-dependent Harrell's C-index in the three models. (D) The DCA for predicting 2-year PFS. The IDI and continuous NRI of the MM-BHAP model compared to ISS stage (E) or R-ISS stage (F).

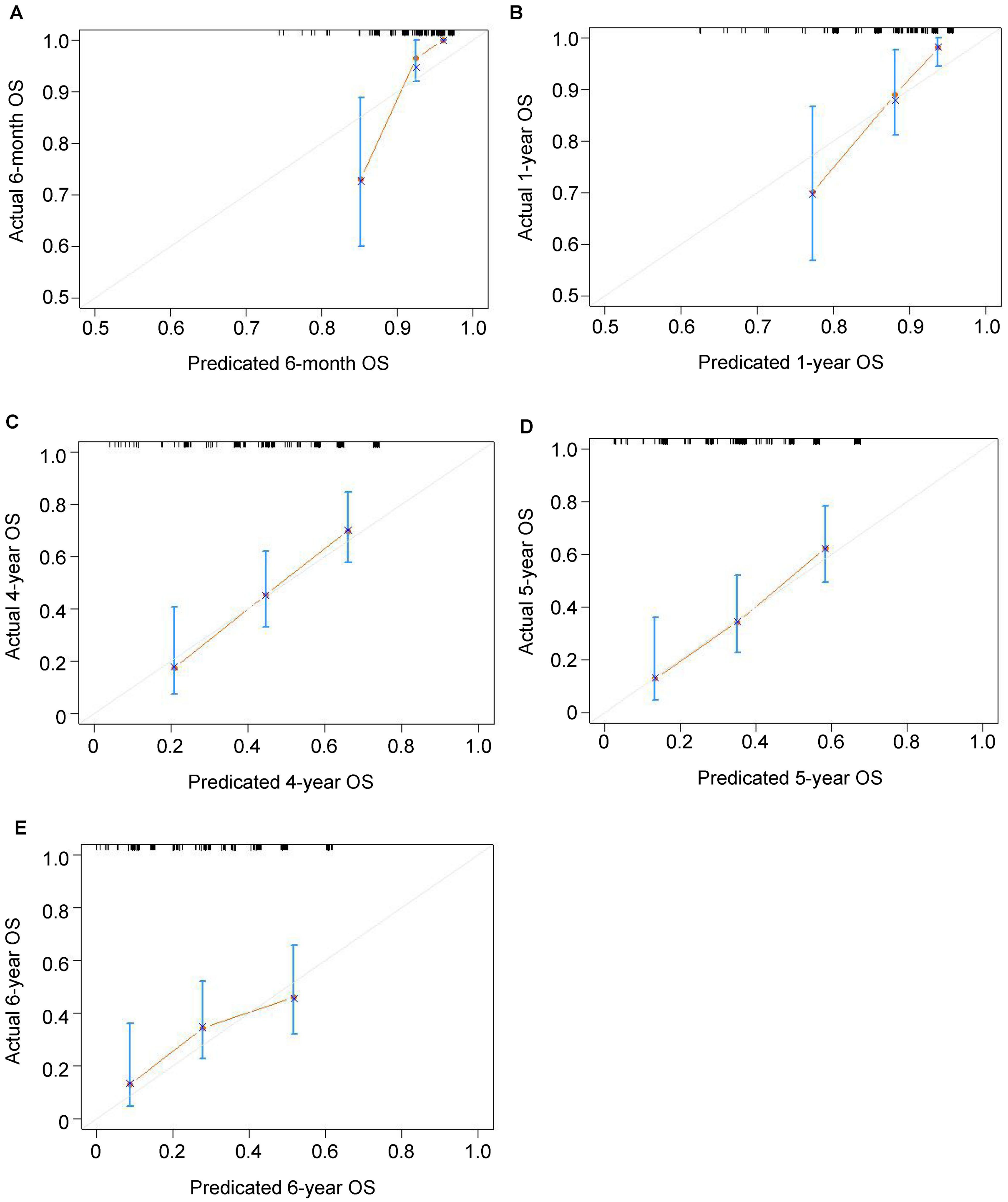

Figure S6. The calibration curve of the MM-BHAP model for predicting 6-month (A), 1-year (B), 4-year (C), 5-year (D), and 6-year (E) OS in the validation cohort.

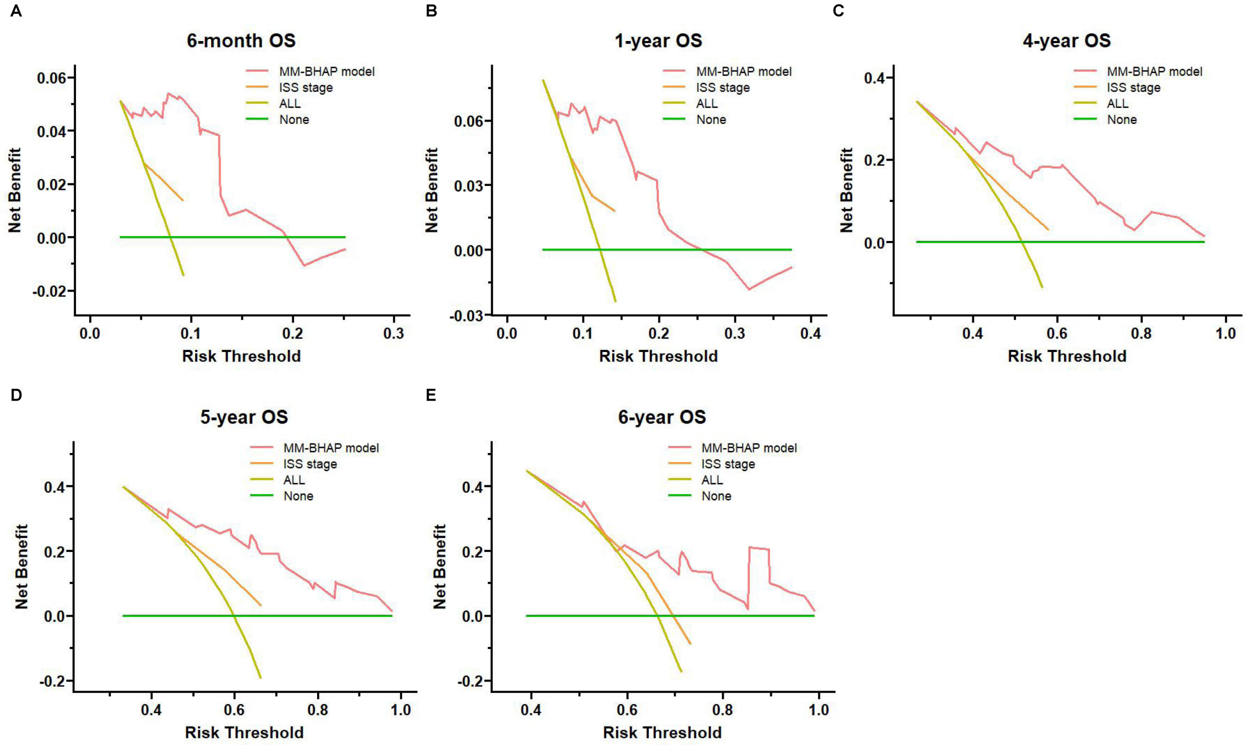

Figure S7. The DCA of the MM-BHAP model and ISS stage for predicting 6-month (A), 1-year (B), 4-year (C), 5-year (D), and 6-year (E) OS in the validation cohort.

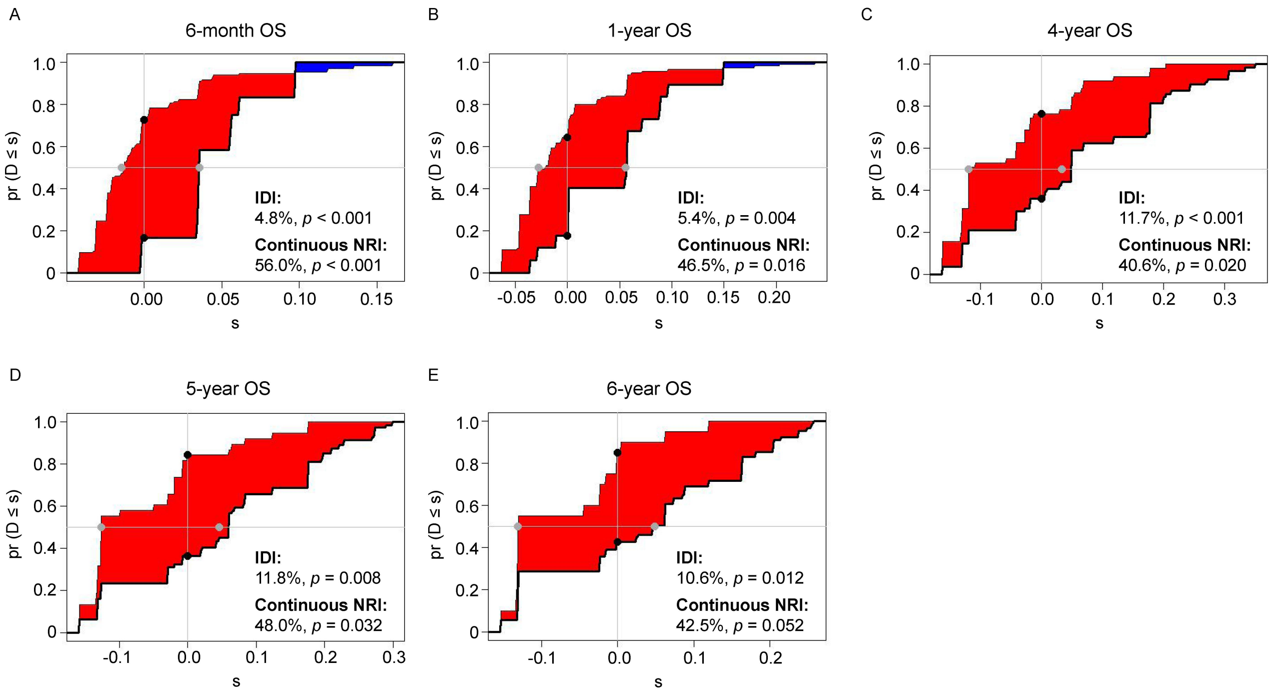

Figure S8. The improvement in prediction of the MM-BHAP model compared to ISS stage for predicting 6-month (A), 1-year (B), 4-year (C), 5year (D), and 6-year (E) OS according to IDI and continuous NRI in the validation cohort.

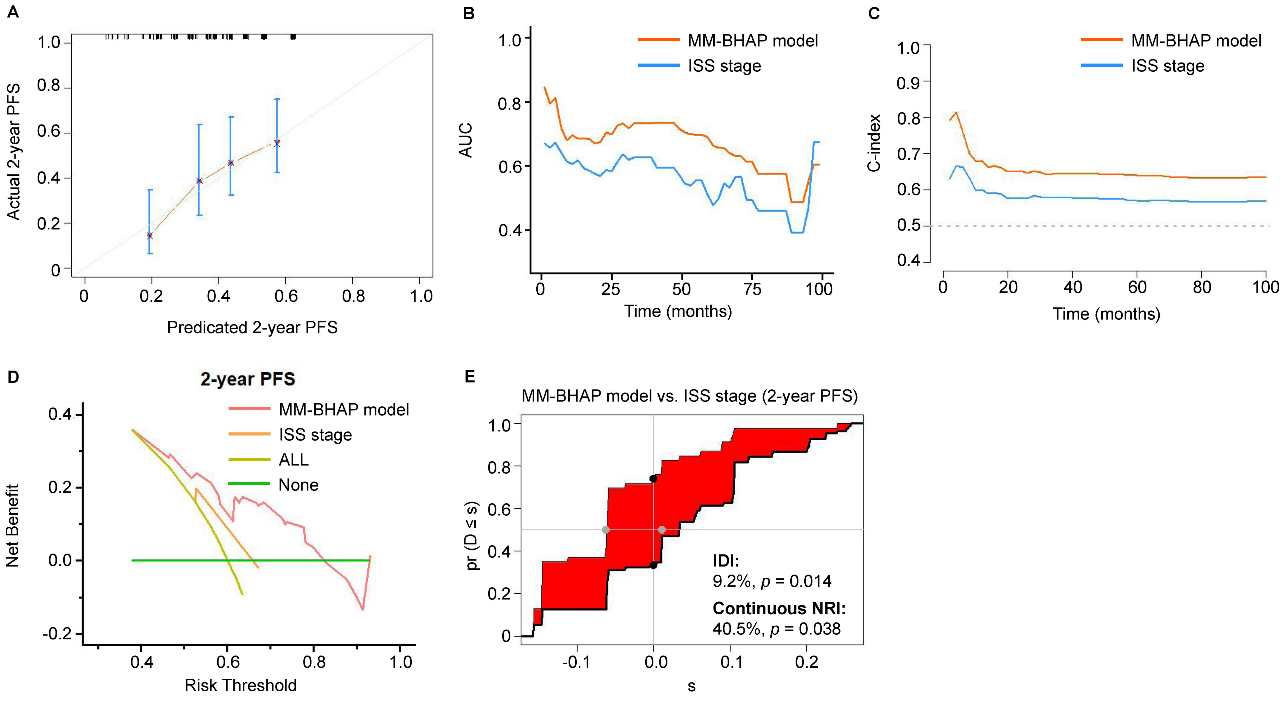

Figure S9. The performance of the MM-BHAP model and ISS stage for predicting PFS in the validation cohort. (A) The calibration curve of the MM-BHAP model for predicting 2-year PFS. (B) The time-dependent AUC of the ROC in the two models. (C) The time-dependent Harrell's C-index in the two models. (D) The DCA for predicting 2-year PFS. (E) The IDI and continuous NRI of the MM-BHAP model compared to ISS stage.
